# Effectiveness of Ball Attachment Systems in Implant Retained- and Supported-Overdentures: A Three- to Five-Year Retrospective Examination

**DOI:** 10.3390/dj7030084

**Published:** 2019-09-01

**Authors:** Luca Ortensi, Matteo Martinolli, Carlo Borromeo, Francesco Mattia Ceruso, Marco Gargari, Erta Xhanari, Marco Tallarico

**Affiliations:** 1 Private Practice, 40123 Bologna, Italy; 2 Private Practice, 45014 Porto Viro, Italy; 3 Private Practice, 20831 Seregno MB, Italy; 4 Department of Dentistry, Fra G.B. Orsenigo-Ospedale San Pietro F.B.F., 00100 Rome, Italy; 5 Department of Clinical Science and Translational Medicine, University of Rome, Tor Vergata, 00100 Rome, Italy; 6 Department of Implantology and Prosthetic Aspects, Aldent University, 1001 Tirana, Albania; 7 Private Practice, 1001 Tirana, Albania; 8 Private Practice, 00151 Rome, Italy

**Keywords:** implant overdenture, metal bar, ball attachments, dental implants

## Abstract

Purpose: To evaluate implant and prosthetic survival rates, complications, patient satisfaction, and biological outcomes of patients rehabilitated with a ball attachment system for implant retained- and supported-overdentures (IOV), which was in function for 3 to 5 years. Methods: This retrospective study evaluated data collected from patients treated between April 2001 and May 2018 with IOV on splinted and non-splinted implants and a ball attachment system. Patients were followed for 36 to 206 months (mean follow-up was 128.1 ± 51.9 months). Data were collected at the 3- and 5-year follow-up examination. Outcome measures were implant and prosthetic survival rates, technical complications, marginal bone loss (MBL), oral health impact profile (OHIP), and periodontal parameters (bleeding on probing and plaque index). Results: A total of 46 patients (16 males and 30 females) with 124 implants were included in this study. Twenty-five implant-retained overdentures were delivered on 53 unsplinted implants, while the other 21 patients received an implant-supported overdentures and the implants were splinted. At the five-year follow-up examination, one implant and one prosthesis failed in the unsplinted group, resulting in a cumulative survival rate of 97.8% at the patient level. Two minor technical complications were experienced. Conclusions: Implant overdenture retained or supported by ball attachment systems showed high implant and prosthetic survival and success rates. A low number of complications, high patient satisfaction, and successful biological parameters were experienced in the mid-term follow-up. Data need to be confirmed by further randomized trials.

## 1. Introduction

Edentulism is defined as “the state of being without any natural permanent teeth. It is an irreversible condition that is evident in age groups of 65 years and older, and was previously considered part of the normal aging process” [1]. To make matters worse, edentulousness is often associated by lower quality of life due to negatively affecting general as well as oral health [2]. Elderly patients could be forced to modify their dietary habits in favor of less fibrous foods, due to an important reduction in masticatory function. Because of this behavior the risk of cardiovascular diseases and gastrointestinal disorders may increase [2]. Phonetic and speech functions are also affected particularly after the loss of anterior teeth, making edentulous patients less confident and limited to interacting with other people [3,4]. Trying to solve these problems, in these cases, dental implants can be invaluable. To overcome the above problems implant-retained and -supported overdentures have been proposed during last decades for restoring completely edentulous patients, as an alternative and more effective treatment modality to the conventional complete removable denture. High long-term success rates and improved patients’ quality of life were reported for implant-retained and -supported overdentures [5,6,7]. Implant-supported overdenture (I-SO) takes the bite force through the implants and into the jawbone, providing the most natural and effective bite for patients. However, treatment is usually more expensive since a greater number of implants are required. With implant retained overdenture (I-RO), the gingiva and the underlining bone absorb the bite force. Fewer dental implants are required, so treatment is more cost-effective and often it may be possible to use mini dental implants.

Various attachment systems have been used for years as retentive elements for root overdentures and are now being used almost exclusively to stabilize an overdenture to the installed as implants, including, but not limiting to, balls, magnets, bars, and telescopic attachments [1]. According to a recent Cochrane Systematic Review, there is no sufficient evidence to determine the true effectiveness of different attachment systems for mandibular overdentures, on patient’s needs and satisfaction, prosthodontic success, maintenance, and costs [8]. Among these, ball attachments are the more simple, commonly used and well-proven attachment systems used for anchorage on both splinted and non-splinted implants [9,10], offering high retentive ability, reduced loading forces along the implants, and aid in correcting disparallelism between the implants. However, their clinical application requires more vertical and buccolingual spaces, potentially encroaching on the tongue space, particularly in tapered arches. In addition, gingival hyperplasia around the attachment system may complicate the plaque control and the hygiene maintenance.

This retrospective study primarily sought to examine the effectiveness of ball attachment systems for implant overdentures in daily practice. Then, if there are some differences when implants were splinted. The study was written according to the STROBE (Strengthening the Reporting of Observational Studies in Epidemiology) guidelines.

## 2. Materials and Methods

This study was designed as an open cohort, retrospective, comparative case series study conducted according to the Declaration of Helsinki of 1975, as revised in 2013. A retrospective chart review of existing data, documents, radiographs, and digital files was performed at one center in Italy to evaluate data collected from fully edentulous patients treated between April 2001 and May 2018. Data analysis was designed to preserve the anonymity of the patients. After considering the study design and protocol, the ethical committee of the University of Aldent declared no objection to this research. Any edentulous patients in at least one arch, aged 18 years or older, that required an implant-based restoration were considered eligible for this study. Completely edentulous patients were considered but only one arch was included in the study. Additional inclusion criteria were a Cawood and Howell class II to VI [11], refusing guided bone reconstruction, and the need of lip support. Exclusion criteria were general contraindications to oral surgery, heavy smoking (≥10 cigarettes/day), immediate post-extractive implants, untreated periodontitis (full-mouth bleeding on probing (BoP), a full-mouth plaque index (PI) of ≤25%), allergy or adverse reactions to the restorative materials, and lack of written informed consent.

One to five submerged implants were placed using a conventional free-hand approach, according to the manufacturer’s guidelines. All the implants were placed in the interforaminal or in the pre-maxillary region. An expert surgeon performed all the surgical and prosthetic procedures. Three types of implants were used during the study period.

**Figure 1:**
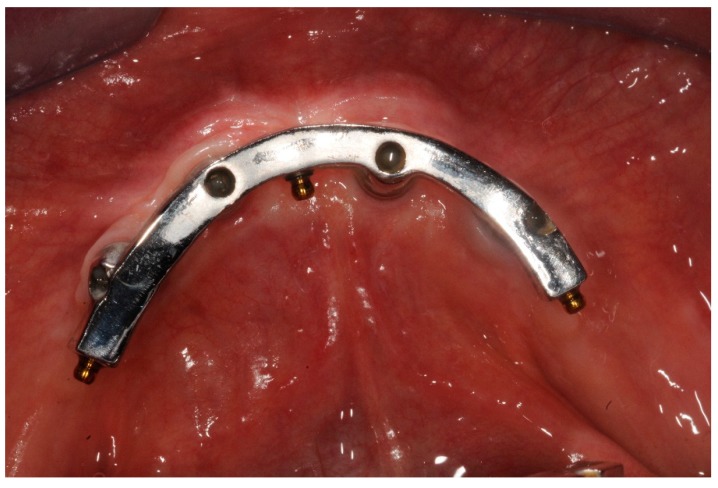
Melted implant-bar, occlusal view at three years follow up.

**Figure 2:**
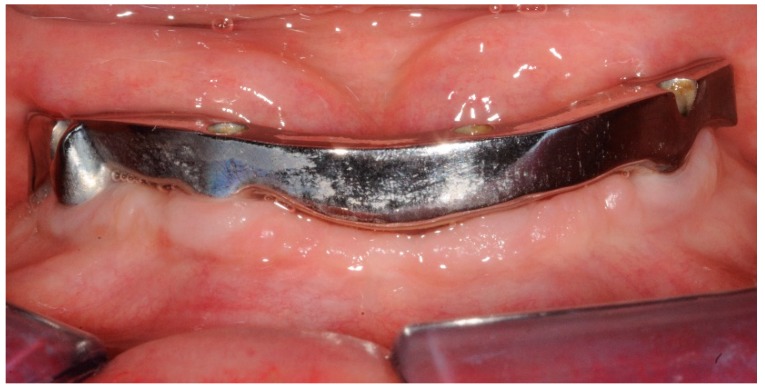
Front view at three years follow up. Good hygiene maintenance and no inflammation of the keratinized mucosa.

**Figure 3:**
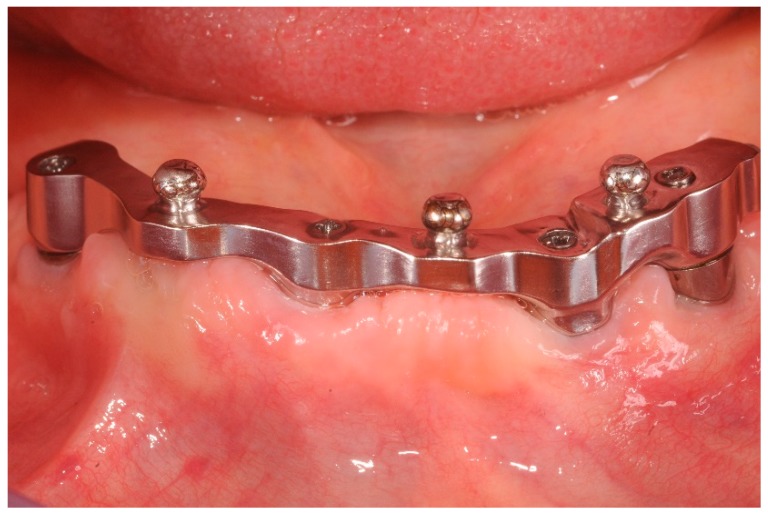
CAD/CAM implant-bar and the metal counterpart, front view at three years follow up.

Three to five months after implant placement, definitive impressions were taken and the models were mounted in a dental articulator in centric relation, using a facial bow, at the established occlusal vertical dimension. Esthetics and function were evaluated and approved by both the patient and the clinician at the try-in appointments. Afterward implant overdentures were delivered. Patients with unsplinted implants received an implant-retained overdenture. A newly developed completely removable denture was delivered on 1 to 5 implants according to a previously published protocol [12]. After delivery of the final prosthesis, all the attachment systems (Ball attachments, OT Cap, Rhein’83, Bologna, Italy) were incorporated chairside into the fitting surface of the overdenture, directly chairside. Patients with splinted implants received an implant-supported overdenture, delivered on 3 to 4 implants. Either the conventional melting technique or newly developed CAD/CAM technologies were used to fabricate the implant-bar and the metal counterpart according to a previously published protocol [13] (Figure 1, Figure 2 and Figure 3). Factors that had influenced the choice between splinted and unsplinted implants were patients’ needs and requests, and the clinician’s recommendation that included health, lifestyle, diet choices, and cost. All the laboratory procedures were accomplished by an expert dental technician (CB). Follow-up visits were scheduled at 1 and 6 months after delivery of the implant overdenture, and then annually. At each follow-up examination, occlusal adjustment was performed if needed. Periapical radiographs were made annually, with a film holder (Rinn XCP, Dentsply, Elgin, IL, USA). The patients were strongly instructed on the daily maintenance hygienic procedures and underwent a professional cleaning by a dental hygienist every 6 months.

## 3. Outcome Measures

Implants and prosthesis failures: An implant was considered a failure if it presented with any mobility, progressive marginal bone loss (annual bone loss of >0.2 mm after the physiological bone remodeling), and suppuration, or any mechanical complications rendering the implant unusable (i.e., implant fracture). A prosthesis was considered a failure if it needed to be replaced with another prosthesis for any reason.

Complications: Any biological (pain, swelling, suppuration, etc.) and/or technical (screw loosening, fracture of the framework and/or the veneering material, etc.) complications were considered. Implants and prosthesis failures and complications were assessed and treated by the treating clinicians at each center.

Marginal bone loss (MBL): Digital periapical radiographs were made with the paralleling technique using commercially available film holders. Mesial and distal bone level changes were measured as the distance from the implant shoulder and the most coronal bone to implant contact, and then averaged. Radiographs were taken at the definitive prosthesis delivery (implant loading) and then yearly. The difference between each follow-up and the baseline was taken as the marginal bone loss. An independent outcome assessor measured all the radiographs using calibrated software (DFW2.8 for Windows, Soredex, Tuusula, Finland).

The Oral Health Impact Profile (OHIP-21) A questionnaire, with 21 questions divided into seven subscales (functional limitations, physical pain, psychological discomfort, physical disability, psychological disability, social disability, and handicap), with two to four questions each, was completed by patients. Patients were instructed to choose from five possible responses ranging from 1 (never) to 5 (very often). The questionnaire was administered by an independent dentist before treatment and yearly after definitive prosthesis delivery.

The bleeding index and plaque index were evaluated yearly around each implant-abutment interface using a periodontal probe (PCPUNC156, Hu-Friedy, Milan, Italy) by an independent blinded dental hygienist. Four sites were evaluated (yes = 1/no = 0) at each implant-abutment complex, and averaged between them.

## 4. Statistical Analysis

All data analysis was carried out according to a pre-established analysis plan using SPSS Statistics for Macintosh (Version 22.0, IBM, Armonk, NY, USA). Descriptive analysis was performed using means, standard deviations, and a 95% confidence interval, as well as median and interquartile ranges (IQR: First quartile; median; third quartile). Fisher’s exact test for count data was used to evaluate statistically significant differences between centers for implant and prosthetic failures and complications. Comparison of the means for OHIP scores between the baseline and the follow-ups was performed by paired tests. Patients were grouped based on their facial type assessment (brachycephalic, dolichocephalic, and mesocephalic) and treated arch (mandible and maxilla). The mean differences in MBL and OHIP between different subgroups were compared using a mixed-model repeated-measures analysis of variance (ANOVA). Fisher’s exact test for count data was used to evaluate statistically significant differences between centers for implant and prosthetic failures and complications.

## 5. Results

A total of 46 patients (16 males and 30 females) with 124 implants were included in this study. Of these, 27 patients were treated in the mandible and 19 in the maxilla. Twenty-five implant-retained overdentures were delivered on 53 unsplinted implants (18 in the mandible and 7 in the maxilla), while the other 21 patients (9 in the mandible and 12 in the maxilla) received an implant-supported overdenture and the implants were splinted. Patients were followed for 36 to 206 months (mean follow-up was 128.1 ± 51.9 months). Data were collected at 3- and 5-year follow-up examinations. Patients’ characteristics were reported in Table 1.

At the three-year follow-up examination, one implant and one prosthesis failed in the unsplinted group, resulting in a cumulative survival rate of 97.8% at the patient level. Two minor technical complications were experienced. The first complication was the detachment of one steel housing in the unsplinted group, and the second was the need to rebase a buccal flange of an implant-supported overdenture, due to food entrapment. Both complications were resolved chairside within 15 to 20 min. At the five-year follow-up examination, no other implants or prostheses failed. Two minor complications were experienced, both in the splinted group. The first complication was the detachment of one steel housing, then the second was the detachment of an upper central incisor. The first complication was resolved chairside in 15 min, while the second was resolved chairside in 60 min.

All the data from 46 patients were analyzed at the 1- and 3-year visit levels, while data from 37 patients were analyzed after 5 years of function (unsplinted, n = 19, and splinted, n = 18). Overall outcome measurements are reported in Table 2.

When comparing data between splinted and unsplinted group, there was no statistically significant difference in all the outcomes measured, including the Oral Health Impact Profile (Table 3), marginal bone loss (Table 4), bleeding on probing (Table 5), and the plaque index (Table 6).

When comparing the number of failed implants, prostheses, and complications between patients with different restorative statuses of the opposing arch, there was no statistically significant difference in all the outcomes measured (*p*-value from 0.6139 to 1.000).

## 6. Discussion

This study aimed to evaluate implant and prosthetic survival rates, any complications, patient satisfaction, and biological outcomes of patients treated with implant overdentures (IOV) and a ball attachment system, on splinted and non-splinted implants, in function for 3 to 5 years. The main limitation of the present study is its retrospective nature, which means there are potentially several biases. Then, because this research was designed as a retrospective cohort study, the clinician should interpret with caution the data that emerged in this paper.

Nowadays, implant-retained or -supported overdentures (IOD) can be considered a viable treatment option increasing masticatory function and improving satisfaction by making up for insufficient retention and stability of a conventional denture [14]. Retention force as well as prosthetic complications of ball and bar attachment systems were evaluated by several studies. Sadowsky [15], in a review of the literature, reported that a single ball attachment allows for less technique sensitive and lower costs compared to other attachment systems. However, ball attachments seem to be less retentive than the bar designed retention. Accordingly, Naert and co-workers [16] showed that a single attachment allow for lower retention compared to a metal bar.

In the present study at the three-year follow-up examination, one implant and one prosthesis failed in the unsplinted group, resulting in a cumulative survival rate of 97.8% at patient level. No statistically significant differences were found between splinted and unsplinted IOV. Recent literature reported successful long-term implant and prosthetic outcomes of patients rehabilitated with an implant-supported overdenture [17,18,19]. The cumulative implant success rate was more than 96% after 15 years of function [17]. Failed implants were commonly experienced in the maxilla, as observed in the present retrospective analysis.

In this retrospective study authors found only two minor technical complications. The first complication was the detachment of one steel housing in the unsplinted group, then the second was the need to rebase a buccal flange of an implant-supported overdenture, due to food entrapment. Both complications were resolved chairside within 15 to 20 min. At the five-year follow-up examination, no other implants or prostheses failed. Two minor complications were experienced, both in the splinted group. The first complication was the detachment of one steel housing, then the second was the detachment of an upper central incisor. The first complication was resolved chairside in 15 min, while the second was resolved chairside in 60 min.

Five years after loading, the mean marginal bone loss observed in the present research was 0.46 ± 0.40 mm, with a minimum of 0.12 mm and a maximum of 2.13 mm. It might be noticed that these results may be in line or even better of the mean marginal bone loss reported by Meijer et al. [20]. The authors report a mean marginal bone loss of 1.0 and 1.1 mm with implant-retained overdenture in function for 5 and 10 years, respectively. According to the results of a systematic review of Cehreli et al. [21], there was no statistically significant difference between splinted or unsplinted implants as well as between different types of attachment systems.

Plaque scores decreased slightly during the follow-up, independently by the number of the implants and the type of attachment systems used. On the other hand, Elsyad et al. [22] reported an increased plaque scores in similar treatments. The authors stated that the reasons could be the resiliency of the attachments, which allows denture movements and accumulation of plaque under the denture. Moreover, age-related problems such as a decreased awareness could affect oral hygiene practice of the patients [23].

It is widely accepted that conventional completely removable dentures have less satisfaction results in patient’s lives compared to IODs [24,25,26,27,28,29]. In the present study, high patient satisfaction was reported, with no statistically significant differences for different IODs design or attachment systems. On the other hand, Tallarico et al., in a long-lasting retrospective study, reported that splinting the implants may reduce the number of mechanical complications. In the same study, Locator attachments showed a higher number of complications compared with other attachment systems [29].

When comparing data between splinted and unsplinted groups, this retrospective study failed to find any statistically significant difference in all the outcomes measured.

## 7. Conclusions

Implant overdenture retained or supported by ball attachment systems showed high implant and prosthetic success rates, a low number of mechanical and biological complications, high patient satisfaction, and good biological parameters, in both the short and mid-term follow-up evaluation. Data need to be confirmed by further randomized trials.

## Figures and Tables

**Table 1:** Patients’ characteristics.

	Total (n = 46)	Unsplinted (n = 25)	Splinted (n = 21)	*p-*Value
Age	69.2 ± 8.1	72.8 ± 7.4	64.8 ± 7.6	0.0008 *
Male	16 (34.8%)	8 (32.0%)	8 (38.1%)	0.7604
Mandible	27 (58.7%)	18 (72.0%)	9 (42.9%)	0.0716
Smokers	7 (15.2%)	3 (12.0%)	4 (19.0%)	0.6857
Mean follow-up (range) in months	128.1 ± 51.9 (36 to 206)	129.2 ± 57.4 (36 to 194)	126.7 ± 45.7 (36 to 206)	0.8689
Mean number of implants	2.7 (1 to 5)	2.1 (1 to 5)	3.4 (2 to 4)	0.0000 *
Failed implants	1	1	0	1.0
Failed prosthesis	1	1	0	1.0
Complications	4	1	3	0.3180

* Statistically significant.

**Table 2:** Overall outcome measurements during follow-up.

	Before *	1 Year *	3 Years *	5 Years ^§^
HOIP	74.04 ± 11.65	32.26 ± 9.21	32.81 ± 7.34	33.0 ± 7.36
Marginal bone loss		0.22 ± 0.30	0.38 ± 0.40	0.46 ± 0.40
Bleeding on probing		0.05 ± 0.10	0.07 ± 0.16	0.09 ± 0.18
Plaque index		0.09 ± 0.15	0.06 ± 0.12	0.07 ± 0.14

* Unsplinted n = 25; splinted n = 21. ^§^ Unsplinted n = 19, splinted n = 18.

**Table 3:** Oral Health Impact Profile.

Group	Before *	1 Year *	3 Years *	5 Years ^§^
Unsplinted	75.0 ± 12.8	32.2 ± 8.6	31.6 ± 6.6	31.4 ± 5.9
Splinted	72.9 ± 10.3	34.5 ± 10.0	34.4 ± 8.1	34.7 ± 8.5
*p*-Value	0.5419	0.4069	0.2467	0.1892

* Unsplinted n = 25; splinted n = 21. ^§^ Unsplinted n = 19, splinted n = 18.

**Table 4:** Marginal bone loss.

Group	1 Year *	3 Years *	5 Years §
Unsplinted	0.20 ± 0.24	0.35 ± 0.37	0.41 ± 0.32
Splinted	0.24 ± 0.36	0.41 ± 0.45	0.51 ± 0.48
*p*-Value	0.6468	0.9931	0.4729

* Unsplinted n = 25; splinted n = 21. ^§^ Unsplinted n = 19, splinted n = 18.

**Table 5:** Bleeding on probing.

Group	1 Year *	3 Years *	5 Years ^§^
Unsplinted	0.04 ± 0.11	0.07 ± 0.13	0.05 ± 0.13
Splinted	0.06 ± 0.09	0.08 ± 0.19	0.08 ± 0.14
*p*-Value	0.5115	0.7286	0.4162

* Unsplinted n = 25; splinted n = 21. ^§^ Unsplinted n = 19, splinted n = 18.

**Table 6:** Plaque index.

Group	1 Year *	3 Years *	5 Years ^§^
Unsplinted	0.09 ± 0.15	0.07 ± 0.17	0.06 ± 0.16
Splinted	0.10 ± 0.06	0.10 ± 0.16	0.11 ± 0.19
*p*-Value	0.7923	0.8350	0.4492

* Unsplinted n = 25; splinted n = 21. ^§^ Unsplinted n = 19, splinted n = 18.
